# Predicting response to HER2 kinase inhibition

**DOI:** 10.18632/oncotarget.3036

**Published:** 2014-12-30

**Authors:** Jeff Settleman

**Affiliations:** Discovery Oncology, Genentech, Inc., South San Francisco, CA

The HER family receptor tyrosine kinases, including EGFR, HER2, HER3, and HER4, have been widely implicated in human cancer [1]. For example, *EGFR* activating mutations are seen in 10-30% of non-small cell lung cancers (NSCLC), *HER2* gene amplification is seen in subsets of breast and gastric cancers, and *HER3* and *HER4* activating mutations have also been detected in various solid tumors [2-4]. Ligand-induced signaling downstream of these activated receptors, primarily via the MAP kinase and PI-3 kinase effectors promotes cell proliferation and survival, thereby driving tumorigenesis [1]. Consequently, substantial effort has been undertaken to discover and develop drugs that selectively target these proteins, including small molecule kinase inhibitors and antibodies directed against their extracellular domains. Such efforts have yielded several FDA approved drugs, including the EGFR kinase inhibitors erlotinib, gefitinib, and afatinib, the HER2 kinase inhibitor lapatinib, the EGFR-directed antibodies cetuximab and panitumumab, and the HER2-targeted antibodies trastuzumab and pertuzumab. Numerous other investigational agents targeting these receptors are currently undergoing clinical evaluation.

In the current era of personalized cancer medicine, biomarkers that can be measured in tumor biopsies and are predictive of response or resistance to these various HER-targeted therapeutics have been vital to their successful clinical development [5]. Thus, EGFR kinase inhibitors are approved for use specifically in the setting of NSCLCs harboring activating *EGFR* mutations, anti- EGFR antibodies are used to treat colorectal cancers that harbor wild-type *KRAS* alleles, and HER2-targeted agents are used in the setting of HER2-positive breast cancers. In addition to these “companion diagnostics”, additional candidate biomarkers associated with sensitivity and resistance to these drugs have emerged from various pre-clinical and clinical studies; for example, mutations in genes encoding components of the PI-3 kinase pathway and expression levels of growth factors for the HER family receptors [6, 7]. The potential clinical utility of these additional biomarkers in guiding treatment decisions is currently undergoing evaluation.

In a recent report in Oncoscience [8], Takagi and co-workers examined the activity of TAK-285, an investigational small molecule dual inhibitor of the EGFR and HER2 kinases, in a panel of breast cancer cell lines. They observed a broad range of sensitivity in a short-term viability assay that was associated with expression levels of the *HER2* and *HER3* genes. Thus, the TAK- 285-sensitive cell lines tended to demonstrate relatively higher levels of *HER2* and *HER3* RNA, as well as protein. Moreover, increased phosphorylation of HER3, which appeared to be mediated by HER2 kinase activity, was also associated with the treatment-sensitive subset of cell lines. They also determined in RNAi studies that HER3 is specifically required to maintain the proliferative potential of TAK-285-sensitive cell lines. A role for EGFR inhibition was excluded in these studies, and the authors conclude that phospho-HER3 levels may be predictive of response to a HER2 inhibitory kinase such as TAK-285.

These findings implicate yet another candidate predictive biomarker that may be useful in stratifying patients to optimize the clinical benefit of a therapeutic that targets the HER family receptors. A few significant considerations arise from these observations. For example, would measurement of phospho-HER3 in tumor biopsies be significantly more predictive of response to TAK- 285 than HER2 levels themselves? While it seems that there may be cases of TAK-285-sensitive cells that lack obvious *HER2* gene amplification, detection of HER2 in breast tumors has been optimized over two decades of study, and so this newly observed “subtype” would have to be prevalent at a significant frequency to justify a novel patient stratification strategy? Along these lines, can phospho-HER3 be measured in tumors with sufficient robustness to constitute a clinical biomarker? Since protein phosphorylation is generally labile in tissues, this could present a significant challenge to clinical translation.

These questions aside, there is certainly growing interest in the role of the activation status of HER3 in determining treatment sensitivity and resistance in a variety of human cancers. Levels of HER3 expression and phosphorylation have been implicated in drug response in a growing number of studies—both as a predictive biomarker and as a candidate therapeutic target.
